# The Microbiota–Gut–Brain Axis and Alzheimer Disease. From Dysbiosis to Neurodegeneration: Focus on the Central Nervous System Glial Cells

**DOI:** 10.3390/jcm10112358

**Published:** 2021-05-27

**Authors:** Maria Grazia Giovannini, Daniele Lana, Chiara Traini, Maria Giuliana Vannucchi

**Affiliations:** 1 Department of Health Sciences, Section of Clinical Pharmacology and Oncology, University of Florence, Viale G. Pieraccini, 6, 50139 Florence, Italy; daniele.lana@unifi.it; 2 Department of Experimental and Clinical Medicine, Research Unit of Histology and Embryology, University of Florence, Viale G. Pieraccini, 6, 50139 Florence, Italy; chiara.traini@unifi.it

**Keywords:** amyloid-β, endotoxin, short chain fatty acids, clasmatodendrosis, cytokines, neurovascular unit, vagus nerve, Toll-like receptor 4

## Abstract

The microbiota–gut system can be thought of as a single unit that interacts with the brain via the “two-way” microbiota–gut–brain axis. Through this axis, a constant interplay mediated by the several products originating from the microbiota guarantees the physiological development and shaping of the gut and the brain. In the present review will be described the modalities through which the microbiota and gut control each other, and the main microbiota products conditioning both local and brain homeostasis. Much evidence has accumulated over the past decade in favor of a significant association between dysbiosis, neuroinflammation and neurodegeneration. Presently, the pathogenetic mechanisms triggered by molecules produced by the altered microbiota, also responsible for the onset and evolution of Alzheimer disease, will be described. Our attention will be focused on the role of astrocytes and microglia. Numerous studies have progressively demonstrated how these glial cells are important to ensure an adequate environment for neuronal activity in healthy conditions. Furthermore, it is becoming evident how both cell types can mediate the onset of neuroinflammation and lead to neurodegeneration when subjected to pathological stimuli. Based on this information, the role of the major microbiota products in shifting the activation profiles of astrocytes and microglia from a healthy to a diseased state will be discussed, focusing on Alzheimer disease pathogenesis.

## 1. The MICROBIOTA–GUT–BRAIN Axis

The gut and its microbiota represent the largest absorption organ, and the largest reservoir of microbes in the human body, respectively. The microbiota consists of almost 10^14^ microorganisms that are mainly bacteria. These are the Gram-positive *Firmicutes* (51% of the population), most of which are *Lactobacilli*, and the Gram-negative *Bacteroidetes* (48%). Physiologically and pathologically the gut and its microbiota can be considered a single system (microbiota–gut), whose interactions give rise to responses that affect the functions in organs and systems of the whole organism. Among the systems involved, the central nervous system (CNS) is in constant communication with the microbiota–gut, through the “two-way” microbiota–gut–brain axis. This interaction involves distant and local networks through neural, immunological, metabolic, and hormonal signaling pathways [1], thus dysfunction at every level of the axis may affect all the other components. It has been shown that brain diseases alter the neurochemistry of the enteric nervous systems (ENS), the functioning of the immune system (IS), and the microbiota itself, using top-to-bottom directional pathways [2,3,4]. In addition, several bottom-to-top directional pathways, activated by microbiota products, are necessary for the correct development and physiological functioning of the brain [5]. Changes in the microbiota composition, the dysbiosis, contribute to several neurodegenerative disorders such as Alzheimer disease (AD) [2,5,6,7,8], Parkinson’s disease (PD) [9], multiple sclerosis (MS) [10], and amyotrophic lateral sclerosis [11].

The focus of the present review will be on the role that microbiota products have on astrocytes and microglia to guarantee an adequate environmental milieu for neuronal activity. When subjected to pathological stimuli, such as those deriving from an altered microbiota, both glia cell types can shift their activation profiles from a healthy to a diseased state, triggering neuroinflammatory mechanisms that cause neurodegenerative effects.

### 1.1. The Microbiota–Gut as a Unique System

As previously mentioned, the microbiota–gut can be considered as a single unit with respect to the microbiota–gut–brain axis. Any effect produced in the CNS depends on the activities resulting from the microbiota and gut continuous interaction. In this interplay, the microbiota has a key role by producing different types of molecules, which are expressed on the surface of the microorganism, or secreted. Of note, also the molecules present on the surface can be physiologically secreted as outer membrane vesicles [12]. The contribution of each bacterial species to the integrity/dysfunction of the gut–brain axis is only partially known.

It has been reported that the microbiota modulates neuronal activity through the production of neurotransmitters or through the modulation of host neurotransmitter catabolism [13]. Different bacteria strains produce neurotransmitters such as catecholamines, GABA, serotonin, and glutamate. The microbiota-derived metabolites, affecting the host IS (see below), influence the behavior of the glial cells in both the ENS and CNS. The Gram-positive bacteria produce short chain fatty acids (SCFAs) that exert a trophic action on the enterocytes, favor the Treg lymphocyte conversion with local anti-inflammatory effects. SCFAs also regulate the serum lipid levels and, crossing the blood–brain barrier (BBB), exert anti-inflammatory activity in the brain. Gram-positive bacteria also metabolize glutamate to γ-aminobutyric acid (GABA) [14], which supports the expression of the anti-inflammatory Toll-like receptors (TLR) 2 and 9 and favors the Treg lymphocyte conversion [15]. Among the Gram-positive bacteria, the *Lactobacilli* generate tryptophan metabolites that stimulate the type-3 innate lymphoid cells (ILC3) to produce interleukin 22 (IL22) (see below). 

The Gram-negative bacteria are the main producers of Aβ prion-like proteins (i.e., a-synuclein) and lipopolysaccharides (LPS), and select pro-inflammatory TLR4. Among the Gram-negative bacteria, *B. subtilis* and *E. choli* produce large amounts of Aβ and LPS [8,14,16,17].

In turn, the gut controls the microbiota though several cell populations. The goblet cells produce mucins and, together with the enterocytes, molecules with antimicrobial properties; the microfold M and dendritic cells convey luminal antigens to the Payer patches and neighbor lymphoid nodes [18]; the ILC3, which produces IL22, plays a major role to guarantee the epithelium integrity, preventing the systemic dissemination of commensal and pathogenic microbes [19,20]; finally, the enteric glial cells actively participate in the maintenance of local homeostasis, playing roles in neuron-to-IS communication, intestinal barrier (IB) integrity, neurotransmitter processing and neuroinflammation [21]. Interestingly, one of the most important targets of the microbiota-derived metabolites are the entero-endocrine cells (EECs), which comprise only 1% of the epithelium, but collectively form the largest endocrine system in mammals. These “primed” cells, acting as chemical sensors, have the capability to trigger further changes in other cells in the microbiota–gut system (e.g., primary afferent neurons and enteric glial cells) by releasing vesicles containing hormones, neurotransmitters and other uncharacterized second messengers [22].

In summary, the microbiota produces several molecules that enter the gut wall and deeply condition the epithelium, affect the IB integrity, the enteric IS, and the ENS function; in turn, the latter influences the microbiota metabolism. Changes in the bacteria stains modify the gut activity and its ability to control the microbiota itself, triggering a vicious circle that reverberates on the brain functionality [19]. Further, the ability of the intestinal cells to handle molecules of bacterial origin explains why and how these molecules have access to the entire organism up to the brain, causing beneficial or pathological effects depending on their properties [23].

### 1.2. Microbiota–Gut System, from Dysbiosis to Neurodegeneration

Dysbiosis is a condition characterized by quantitative and qualitative changes in the microbiota composition, of which the results are possibly noxious to the host. The quantitative changes consist in the presence of fewer beneficial microbiota, and the qualitative in a lower variety of microbiota species [24]. The main genera of hostile bacteria are the *Enterobacteriacee*, a family including the gut commensals *Escherichia, Shigella, Proteus*, and *Klebsiella*. Dysbiosis may promote an inflammatory condition because of i) a decrease in the anti-inflammatory bacterial populations (*Lactobacillus* and *Bifidobacterium*), ii) the excessive production of noxious molecules, or iii) IB and BBB dysfunctions. Therefore, dysbiosis might cause an excessive production of noxious molecules, IB and BBB dysfunctions, and the development of several gut and brain disorders [2,5,8,12,25]. The microbiota changes spontaneously through life and during aging the ratio between Gram-positive and Gram-negative bacteria inverts. It remains to be determined how dysbiosis contributes to neurodegeneration and/or vice versa. Though, the accumulated evidence demonstrates a significant association between dysbiosis and the development of neuroinflammation and neurodegeneration. Indeed, in several neurodegenerative diseases a consistent decrease in SCFA [26], high levels of Aβ and LPS (AD brain) [27], and low levels of GABA have been reported [14]. The LPS of microbiota–gut origin, as well as infiltrating lymphocytes, were found in the brains of Alzheimer patients [28,29]. Finally, the patients effected by neurodegeneration showed dysbiosis [30,31,32], while APP/PS1 transgenic mice, who overproduce Aβ, harbor altered microbiota [33].

The published data also show that probiotic supplementation rich in Gram-positive bacteria improves cognition in patients with AD [34], and diet has been proved to prevent or reduce the risk of developing cognitive impairment in animals and humans [35,36,37,38]. In the animal, a long-lasting high-fat diet induces cerebral amyloidosis, commensurate with dietary-induced hyperlipidemia and with an increase in chylomicrons (CM) concentration; starvation reduces the formation of Aβ in the intestine [39,40]. In humans, high-fat and cholesterol-rich diets increase AD risk [41], while Mediterranean and Asian diets may protect against cognitive decline and delay the onset of AD [36].

Attempts have been made to identify those alterations in the microbiota–gut system that could predispose or favor the development of neuroinflammation and neurodegeneration. An interesting hypothesis has recently been formulated on this topic (see insert). It highlights the main role of the microbiota–gut system and indicates it as the privileged target of interventions aimed at preventing the appearance of neurodegenerative diseases or, at least, at slowing down their evolution.

## 2. The DYSBIOSIS and the ALZHEIMER DISEASE

Increased lifespan has resulted in the increased frequency of age-related diseases, including AD, the most common type of dementia accounting for more than 65% of all dementia cases. AD currently affects approximately 40 million aged people in Western countries. The increased life expectancy in the world population has seen a progressive increment of this type of dementia and it is expected to triplicate in incidence by 2050. Indeed, beyond the familial forms of AD, at relatively early onset, the idiopathic and most common forms of AD have late onset and are often indicated with the acronym LOAD (late onset AD). AD is a neurodegenerative pathology characterized by a slow, irreversible decline in the cognitive functions that affect different brain regions. To date, there are no effective pharmacologic agents to prevent or slow down the disease progression.

Since 2010, it was raised the question if AD depends on aging or, instead, the late age allows the disease to clinically manifest as the result of the accumulation of stress factors through the lifetime [42]. Among the identified factors, the alterations in the gut microbiota, and the subsequent inflammatory processes, have been considered responsible for the later (15–20 years) appearance of neurodegeneration [43,44].

### 2.1. The Origin of Amyloid Beta

The histopathological hallmark of AD is the accumulation in the brain of misfolded Aβ peptides that organize in fibrils and deposit in plaques [45,46]. The origin of Aβ has not been clearly established. The literature has mainly focused on the Aβ produced in the brain [47], hypothesizing that Aβ is formed in brain neurons and, with cholesterol and ApoE derived from astrocytes or via the BBB, is embedded in vesicles for further processing and clearance. 

Several studies have shown that, besides the brain, the microbiota–gut system is a site of Aβ production, and that the enterocytes contain substantial amounts of Aβ [40,48,49], and that diet and intestinal microbiota regulate the Aβ presence in the gut epithelium [17,25]. Gram-negative bacteria are a significant source of Aβ and of LPS, and the increased levels of these molecules found in AD brain plaques were related to dysbiosis. Thus, Gram-negative bacteria are likely to be involved in the pathogenesis of neurodegeneration [12,50]. In the attempt to answer to the question whether the Aβ accumulated in the brain is of bacterial or cellular origin, several reports suggest the possibility that bacterial amyloid, which has prion-like properties, would induce molecular mimicry mechanisms and the accumulation of neuronal Aβ into the brain, promoting neuroinflammation and neurodegeneration [8,49,51,52,53,54]. The further question could be as follows: how does Aβ as well as LPS reach the brain? Two different routes have been considered. The first begins from the enterocytes, where Aβ and LPS are integrated in CM-containing ApoE proteins [12,17,48] and, through the blood stream, they reach the brain [45,55]. For Aβ, the following second route of diffusion has been postulated: because of its prion-like proteins, Aβ could arrive to the brain via neuron-to-neuron retrograde transport from the ENS to the brain through the vagus nerve [25,56,57]. This retrograde neuronal pathway was already described for α-synuclein in Parkinson’s disease [8,56]. When Aβ reaches the brain, local chaperonins, and the receptors for advanced glycosylation products (RAGE) are debuted to their clearance through the BBB. The overload or defective clearance of Aβ may cause its accumulation, favoring fibrils organization and their deposition. Similarly, high levels of LPS increase the BBB permeability, enter the brain, and activate several inflammatory pathways [12,58]. Recently, some amino acids, such as isoleucine and phenylalanine, have also gained importance in AD pathogenesis. It has been reported that these amino acids drive neuroinflammation during AD progression through stimulating the differentiation and proliferation of pro-inflammatory T helper 1 (Th1) cells [59]. 

### 2.2. AD, Dysbiosis and Metabolic Diseases

Dysbiosis is constantly associated with insulin resistance and type 2 diabetes, dyslipidemia, hypertension, obesity and, because of the significant association found between metabolic diseases and AD, the formers are considered as possible etiological factors for AD [24,34,49,60]. Among the taxa present in the microbiota, it has been shown that, in dysbiosis, the imbalance between *Proteobacteria* and *Bifidobacteria* in favor of the former is associated with a decrease in the synthesis of SCFAs and the appearance of dyslipidemia. *Bifidobacteria* owns lipid-lowering and hypocholesterolemic activity, facilitating the fecal elimination of cholesterol and reducing its absorption. This taxon also promotes an increase in the serum levels of leptin, an anti-obesity hormone [24,61]. The observation that statin treatment for hypercholesterolemia was associated with a lower risk of developing AD, and the high frequency of elevated plasma level of this lipid in AD patients, has moved the attention to the implication of cholesterol and its metabolites in the pathogenesis of AD [62]. Altered cholesterol metabolism deeply affects the bile acids (BAs) production and metabolism [50]. BAs are synthesized in the liver from the cholesterol, and their blood levels as well the type of BA present depend on a combination of liver and microbiota co-metabolism [50,63]. Studies in AD patients have demonstrated a significant association between an altered BA profile in the blood and cognitive impairment [63]. Thus, dysbiosis triggers several changes in the organ functions, all conducible to an inflammatory status powered by the guest hostile bacterial strains [14,24,35,50,54].

## 3. The MICROBIOTA and the CENTRAL NERVOUS SYSTEM GLIAL CELLS

The contribution of the different bacterial strains to the integrity and dysfunction of the microbiota–gut–brain axis is not completely known. 

Some brain areas, such as the cortex, hippocampus, and amygdala, are particularly susceptible to the products of the microbiota [5], and these areas correspond to those primarily altered in AD. Although brain diseases were traditionally attributed solely to the malfunctioning of neurons, it is becoming more and more evident that proper interplays among neurons, astrocytes, microglia with peripherally derived cells and molecules are of fundamental importance for the physio-pathological organization of the brain [64]. Nevertheless, recently a fourth actor has come into focus, the microbiota, which releases factors that are fundamental for the physiological functionality of astrocytes and microglia. Alterations of the microbiota can reverberate on the cells of the CNS, and particularly on the astrocytes and microglia, modifying their functions. Much remains to be explored regarding the involvement of the different microbial taxa, of other peripherally derived cells, and of molecules that regulate the microglia and astrocyte functions. The microglia and astrocytes can have simultaneously multiple profiles of activation, which can represent the extremes of a continuous spectrum of reactive profiles [65]. The mechanisms regulating their diverse functional properties remain unknown, but evidence suggests that environmental cues, such as those deriving from the microbiota, are important not only in physiological conditions, but also in many neurodegenerative diseases such as AD.

The following paragraphs of the review will delineate the current knowledge on how microbiota regulates the physiological and pathological functions of astrocytes and microglia, to assess how these interactions can influence the disease state and its progression.

### 3.1. Physiological Functions of Astrocytes and Their Microbiota-Driven Alterations

Astrocytes, the most numerous glia cells of the CNS, are endowed of many housekeeping functions and help to maintain the brain in healthy conditions [66,67,68,69,70]. Among other functions, astrocytes are an integral part of the BBB, of the neurovascular unit (NVU), and of the glymphatic system, and they regulate neurovascular coupling, vascular tone, and blood flow [66,69,71,72], and, together with perivascular microglia and macrophages, survey the influx end efflux of molecules [73]. Disruption of the NVU is associated with vascular dementia [74] and increased permeability of the BBB has been observed in subjects with mild cognitive impairment [75], likely contributing to the early stages of AD [76], as also shown in animal models of the disease [77,78,79].

Astrocytes, in their activated form (A1 astrocytes), express and release cytokines that modify the permeability of the BBB [80], and the activation (astrogliosis) of perivascular astrocytes causes the loss of aquaporin4 (AQP4) polarization and may cause vascular and glymphatic dysregulation and BBB disorganization, considered among the first steps in AD pathogenesis [78,81,82]. Astrocytes during aging undergo a morphological and functional modification named clasmatodendrosis [83,84,85], which consists of the fragmentation and shortening of the astrocytes distal processes. Clasmatodendrosis is associated with changes in cell function [86], which can compromise the integrity of the BBB [87], and possibly of the NVU and the glymphatic system, which is impaired during aging [88]. BBB, NVU and glymphatic system dysfunctions are involved in many neurodegenerative disorders, particularly those in which the accumulation of extracellular waste is an important characteristic. Therefore, clasmatodendrosis can hamper astrocyte-mediated Aβ clearance from neurons and increase fibrillar Aβ deposition [89,90]. The deposition of high quantities of fibrillar Aβ modifies the interactions between the astrocytes and neurons [90], possibly decreasing Aβ peptide disposal to the circulating system, further increasing Aβ deposition in the brain parenchyma [91], which can have a significant role in neuronal damage. In mouse models of AD, the impairment of Aβ clearance increases neurodegeneration [92].

All the above functions of astrocytes can be altered when the microbiota is modified, in both healthy and disease conditions. It has been demonstrated that the endothelial cells of germ-free (GF) mice have decreased expression of occludin and claudin-5, with consequent disorganization of tight junctions and increased permeability of the colonic barrier [93] and of the BBB [94]. The products of the microbiota, mainly butyrate, maintain the integrity of these barriers [95]. Indeed, in GF mice the recolonization of the gut with microbiota increases the expression of tight junction proteins and promotes the restoration of BBB integrity [94]. Further, the supplementation of GF mice with SCFAs restores the BBB [96]. In addition, molecules of bacterial origin, such as LPS, induce the transcription of the proinflammatory and cytotoxic pathways in astrocytes [97] and the breakdown of inter-cellular tight junctions [98], which causes further structural and functional alterations of the BBB. The BBB, separating the CNS from the periphery, maintains a milieu that is required for the proper functional activity of neurons and neuronal circuits [99,100]. Therefore, alterations of the BBB, such as those caused by dysbiosis, allow the passage of proinflammatory factors, of immune cells from the periphery and of peptides such as Aβ, and modify the composition of the cerebral milieu and the homeostasis of the brain cells. In a further study, it has been shown that during treatment with antibiotics, while in the hippocampus the expression of tight junction proteins decreases, and in the amygdala, it increases [101]. This region-specific differentiation of BBB permeability possibly can result in differential passages of molecules in the various regions of the brain, with differential effects. It is still to be understood the causes of the diverse spatial responses of the different brain areas to peripheral stimuli.

The disruption of the BBB, NVU and of the glymphatic system causes a reduction in the transport and inefficient removal of toxic substances, which can accumulate in the brain parenchyma, implementing a vicious circle of neuroinflammation and tissue damage [80,102,103,104,105,106]. Since the glymphatic system facilitates the clearance of interstitial Aβ and tau [107], the impairment of all these mechanisms decreases Aβ clearance [88,108,109], increasing Aβ extracellular levels. All these data suggest that the modifications of astrocytes functionality, caused by dysbiosis, are responsible for microlesions of the NVU and of the glymphatic system, decreasing the disposal of Aβ peptides in the brain parenchyma, and increasing the risk of amyloid plaque formation [107].

### 3.2. Microbial Products That Shape Astrocytes

While it is known that several factors within or outside the CNS cause the activation of astrocytes from their healthy state, much remains to be explored with regards to the microbial taxa that finely regulate astrocyte functions. Microbial-derived products and metabolic by-products (SCFAs) activate distinct immune pathways in the host. The gut microbiota can modulate the activity of astrocytes metabolizing dietary tryptophan to produce natural ligands for aryl hydrocarbon receptors (AHRs), including indole-3-aldehyde and indole-3-propionic acid, which bind to astrocyte AHR [20,96,110]. It has been demonstrated that indole-3-aldehyde treatment reduces the expression of proinflammatory factors [96]. Furthermore, the upregulation of AHRs in astrocytes results in anti-inflammatory activity through interferon-I (IFN-I) signaling [96]. It appears that IFN-I works with microbiota-produced dietary tryptophan to activate the AHRs in astrocytes and to suppress inflammatory mechanisms [96]. Collectively, these findings suggest that the microbial metabolites of dietary tryptophan can modulate the inflammatory status of astrocytes, with important consequences for neuroinflammation. The involvement of peripheral bacteria in brain health and disease conditions can be also envisaged by other data. Indeed, *Porphyromonas gingivalis*, one of the most common Gram-negative bacteria in oral chronic inflammatory diseases, activates astrocytes via TLR4, thus increasing cytokine production and contributing to the inflammatory lesions [111,112].

### 3.3. How the Gut Microbiota Shapes Microglia

In physiological conditions microglia survey the brain parenchyma, maintain microenvironmental tissue homeostasis [113], perform pruning of synapses or phagocytosis of apoptotic neurons and debris, and maintain astrocyte functions. The regulation of neuronal activity (activation, inhibition, potentiation, or depression) has been viewed for over a century as an exclusive prerogative of neurons themselves. Nevertheless, recent data demonstrate that even microglia can be involved in this process, acting similarly to inhibitory neurons to suppress excessive neuronal activity [114,115,116,117,118,119], at least in the striatum, but possibly also in other brain regions.

Microglia are myeloid cells that invade the brain during early development and have dynamic roles in the coordination of responses between the immunity system and cognitive functions [120,121,122,123,124,125,126,127,128,129]. Microglia are the primary immune cells of the CNS, and being active responders to peripheral stimuli, the standard notion that the brain is an “immune privileged” organ is rapidly changing [130]. Indeed, various pathological stimuli cause the rapid recruitment of microglia to the site of injury, resulting in a resident innate immune response [113,131,132]. The dysfunction of microglia has been described in many CNS disorders, such as AD [133], frontotemporal dementia [134,135] and PD [136]. The ability of microglia to maintain their protective role by clearing dying neurons [137] decreases considerably in a proinflammatory context [138]. For instance, in APP-SL70 mice, a transgenic model of AD, microglia phagocytic activity inversely correlates with Aβ plaque deposition and aging [139].

Microglia are highly dynamic cells, and their highly mobile projections are necessary to sense the domains of neighboring microglia cells to avoid their spatial overlap [140,141]. In the absence of microbiota, such as in GF or specific pathogen-free (SPF) mice, microglia morphology is severely altered, and the cells have longer, very mobile, hyper-ramified projections [142,143,144], which enter in physical contacts with the projections of adjacent cells and partially overlap in the spatial domains of neighboring microglia [142]. In GF or SPF mice treated with antibiotics, the microglia phenotype returns to normal [142]. The consequences of the hyper-ramified microglia phenotype in microbiota-free animals are not clear, although recent research shows that microglia prune synapses in a microbiota-dependent manner [145], which indicates a possible functional consequence of the alterations of microglia projections.

As discussed above, an emerging hypothesis is that the microbiota influences AD pathology, increasing Aβ production in the gut, which may cause increased Aβ deposition in the brain, Aβ plaque formation and activation of microglia. The activated microglia migrate to the sites of Aβ plaques, interact with Aβ deposits and regulate Aβ levels in the brain [146,147]. Germ-free APP/PS1 mice (a transgenic model of AD) have a drastic reduction in Aβ levels and of compact Aβ plaques, as well as decrease in IBA1-positive microglia, in comparison to APP/PS1 mice with normal microbiota [33]. Therefore, it appears that signals from the microbiota delineate microglia morphology and functionality, and dysbiosis causes microglia dysfunctionality. Erny and coworkers [142] demonstrated that the microbiota is important for the maturation and maintenance of microglia in proper steady-state physiological conditions, ready to display a rapid response to damaging stimuli. The reconstruction of microbiota reverses, although not completely, the microglia cell morphology [142]. Nevertheless, how the gut microbiota can control microglia maturation at such distant sites, such as the CNS, remains to be unraveled.

In the last decade it has become clear that microglia respond to noxious stimuli integrating multifarious inputs and their responses can be opposite and, depending upon the stimulus, can induce neuroprotective or neurotoxic effects. Indeed, the original definition of microglia activation has been revised on the demonstration that microglia can assume at least two different phenotypic forms, M1 and M2 [148,149,150]. It is now clear that M1 and M2 represent the two extremes of an entire spectrum of activation patterns. M1 microglia express proinflammatory cytokines such as IL-6 and TNF-α. M2 microglia express high levels of arginase-1 (Arg-1) and IL-10 [149,150]. The M2 phenotype is thus more active in the phagocytosis of apoptotic or dying neurons to prevent secondary inflammatory mechanisms and promote tissue regeneration [64,151,152]. Furthermore, phagocytic microglia are classified into M2a, M2b and M2c in the absence of inflammation, and they induce a Th2-like response [153]. In the hippocampus, microglia have a high “immune-vigilant” phenotype, which can be responsible for the higher microglia activation in response to Aβ plaque formation, giving rise to a harmful chronic inflammatory response [154]. Hart et al. (2012) [155] showed a further regional difference between the microglia located in the white matter versus the microglia located in the grey matter [155]. In a different study it was demonstrated that hippocampal microglia display lower expression of many proteins, among which is CXCR3 [156], a receptor involved in neuron–microglia communication, in microglia recruitment, neuronal reorganization [157], and in microglia activation during demyelination [158]. Therefore, decreased levels of the CXCR3 receptor and other proteins in AD-vulnerable brain regions, such as the hippocampus, may impair the microglia response and recruitment. Consequently, region-specific variations (both increase or decrease) in gene expression may be implicated in the progression of neurodegenerative diseases [159].

The physiological interactions of M1/M2 can be compromised in brain pathologies, and microglia often actively participates in disease progression. In AD, microglia, activated by danger signals such as ATP released from dying neurons, retract their branched processes, round up, produce IL-1β, TNF-α, ROS and NO, thus contributing to the amplification of inflammation and neurodegeneration. Indeed, the possibility to control and modify the microglia phenotype represents a challenge to contrast this disease. The host microbiota is an essential environmental factor that shapes the brain’s innate IS, and particularly the maturation and function of the microglia [142].

### 3.4. Microbial Products That Shape Microglia

Bacterial-produced molecules, such as LPS, peptidoglycans and PAMPs (pathogen-associated molecular patterns) [160], can cross both the IB and the BBB [161,162] and can reach the brain parenchyma where they can be recognized by TLR4 expressed on microglia, which plays an important role in neuroinflammation [163]. Further, the stimulation of TLR2 by fibrillar Aβ activates microglia into a more pro-inflammatory profile, with detrimental effects on AD pathology [164]. Nevertheless, although microglia actively maintain their protective role during normal aging [132,165,166,167], by clearing dying neurons [137], their capability is considerably impaired in acute pro-inflammatory contexts. Furthermore, the sustained activation of microglia can increase Aβ deposition and phagocytosis of healthy neurons [168,169,170,171,172], thus intensifying neurodegeneration [173,174].

Therefore, the microbiota shapes the brain’s innate IS, conditioning the maturation and function of microglia [142], which, in turn, has a dynamic role in coordinating the responses between the IS and cognitive functions [122,123,129]. It is thus conceivable that the modifications of the microglia phenotypes during their activation (such as downregulation of P2RY12 and other receptors) can contribute to pathological dysfunctions of neuron excitability and, consequently, behavioral alterations typical of neurodegenerative disorders such as AD [175,176,177,178].

**Figure 1:**
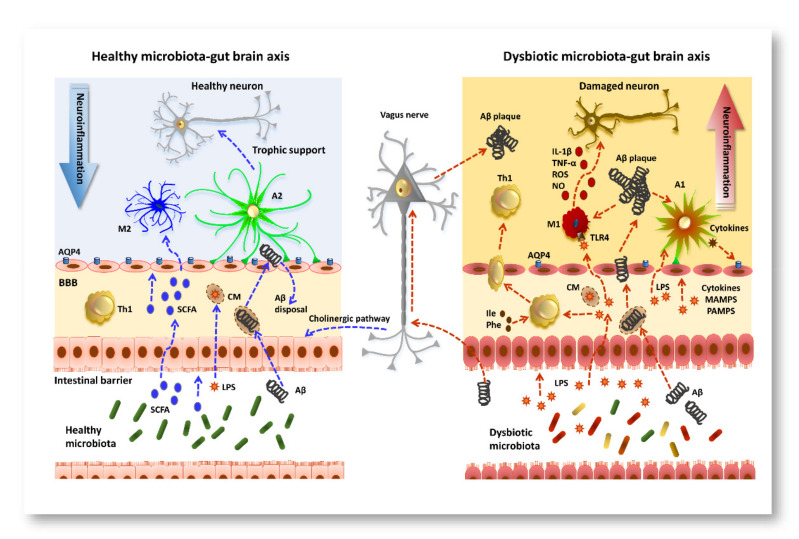
Schematic representation of the bottom-to-top regulation of neuroinflammation in AD pathogenesis. (**Left panel**) In healthy conditions, the microbiota–gut–brain axis modulates key processes, including immune cell maturation and maintenance of the gut epithelium. SCFAs produced by the gut microbiota cross the IB and, via the circulatory system, reach and cross the blood–brain barrier. Once in the brain parenchyma, SCFAs target microglia and regulate their functions. The gut microbiota is one of the main producers of Aβ peptide and LPS, which integrate in CM and cross the BBB. Aβ is readily retro transported to the circulatory system for its disposal. Blue lines represent known beneficial pathways of microbiota. (**Right panel**) Microbiota overproduction of LPS and cytokines causes modification of the permeability of the gut epithelium, of the NVU and of the glymphatic system. Gut microbiota production of SCFA is reduced in AD, while the production of proinflammatory cytokines, including IL-1β, IL-6, and TNF-α, as well as MAMPs and PAMPs, is increased. These factors translocate to the brain where they modulate microglia via TLR4, and activated M1 microglia release IL-1β, TNF-α, ROS, and NO that cause neuronal damage. In addition, proinflammatory cytokines cause activation of astrocytes (A1), which release cytokines that in turn decrease AQP4 expression and modify NVU permeability. Peripheral Th1, activated by isoleucine (Ile) and phenylalanine (Phe) produced by microbiota, can recognize the bacterial metabolites or MAMPs and migrate to the brain via the damaged BBB. Aβ peptide produced by the microbiota can easily cross the IB or be retrogradely transported to the brain via the vagus nerve. Since disposal of Aβ peptide is impaired by the damage to the BBB, Aβ peptide precipitates to form plaques, which further worsens microgliosis and astrogliosis, increasing the severity of AD pathology. Red lines indicate the bottom-to-top damaging pathways so far demonstrated.

Many hypotheses have been postulated to explain how the microbiota can regulate microglia. I) SCFAs generated by the microbiota can cross the BBB. Once in the CNS, SCFAs target the microglia and regulate their function or maturation. II) Immune cells expressing receptors for SCFAs, after interacting with SCFAs, can migrate to the brain via the BBB. III) Before the expression of SCFA-recognizing receptors, other metabolites or compounds called microbe-associated molecular patterns (MAMPs), produced by the microbiota, can cross the BBB, and target the microglia to regulate their function or maturation. IV) Peripheral macrophages that can recognize MAMPs released by the gut microbiota can migrate to the brain and cross the BBB. V) Finally, the gut microbiota can communicate directly with the CNS resident microglia through the vagus nerve [179]. The vagus nerve senses changes in proinflammatory cytokines caused by inflammation in the gut, and through its afferent fibers sends information to the CNS and influences microglia and inflammatory mechanisms [180]. Most of the above mechanisms take advantage of the disruption of the BBB or the glymphatic system, described in the previous paragraph (see Figure 1).

Furthermore, epigenetic mechanisms can shape the identity of macrophages during development, but the local microenvironment within and outside the brain can additionally reprogram the genetic imprint [142,181,182]. Although little is known on the epigenetic mechanisms that control the function/activation of microglia, prenatal ablation of histone deacetylases1/2 (HDAC1/2) impairs microglia development, while it has no effect on microglia homeostasis in adult mice [183]. Interestingly, in a mouse model of AD, a deficiency of HDAC1/2 in microglia increases amyloid phagocytosis, resulting in decreased Aβ load and amelioration of cognitive impairment [183]. It appears therefore that epigenetic factors, which can have different outputs whether during development or in adulthood, affect microglia maturation, homeostasis, and activation in a differential manner. The gut microbiota can affect epigenetic modifications throughout the entire lifespan, as has been demonstrated in diabetes and obesity [184], but possibly also in AD.

Recently it has been shown in APP/PS1 mice that a dynamic shift in gut microbiota composition is significantly correlated with the increase in the Th1 cells infiltration into the brain [54]. The ablation of the gut microbiota by antibiotics blocks Th1 cells infiltration and M1 microglia activation. These findings highlight that gut microbiota is a driving factor in promoting Th1/M1 microglia neuroinflammation in AD progression [54].

## 4. Conclusions

In this review we have summarized the role of the microbiota–gut–brain axis as an integral part of the pathogenesis of AD. Indeed, the “two-way” interactions among the intestinal microbiota, the peripheral immune system, and the CNS are essential for the maintenance of the host’s health, and their dysregulation can be one of the initiating factors in multifactorial chronic neuroinflammatory diseases, such as AD. Neuronal pathways, hormones, microbial molecules, and metabolites are all involved in the signaling between these two regions. Although the causes of AD are still not clear, and no curative treatments are available, the experimental and clinical data collected strongly address the research versus preventive approaches aimed at reducing Aβ production and/or inhibiting the self-assembly of amyloidogenic peptides. The modification of the composition of the microbiota destroys the bottom-to-top communication that ultimately influences brain motor, sensory, and cognitive functions, maintains brain homeostasis and/or contributes to the onset of pathological conditions. Elucidating the interplay between the gut microbiota and the central nervous system, and the role of the microbiota in neuroinflammation, will lead to a better understanding of many neurodegenerative diseases pathogeneses.

Human-induced pluripotent stem cell (iPSC) technology, a recent bioengineering technique, is considered a promising methodology to reproduce, in vitro, complex systems such as the microbiota–gut–brain and to connect them to each other. Further, iPSC can be utilized to differentiate all major brain cell types to study many neurodegenerative diseases [185,186,187]. Using iPSC-derived cells from normal and diseased patients, it is now possible to understand the complex cellular/molecular interplay that occurs between the different brain cell types in AD. It can be envisaged that this new technology can be of importance to understand the complex communication between the microbiota and brain cells, recapitulating the bottom-to-top directional pathway in a simpler system that can also be used to generate organoids that mimic native brains [186].

A special mention is deserved by the MINERVA platform (MIcrobiota–Gut–BraiN EngineeRed platform for eVAluating the impact of intestinal microflora on brain function) founded by the European Research Council (ERC) [188]. MINERVA is designed to allow researchers to develop therapeutic strategies using a personalized medicine approach. A deeper knowledge of microbiota–gut–brain interactions may lead to new therapeutic approaches through which neuroinflammation/neurodegeneration can be dampened, acting indirectly through the microbiota–gut–brain axis. Notably and interestingly, the administration of sodium oligomannate (GV-971), a mixture of oligosaccharides, has been shown to reduce the levels of these amino acids in the blood and brain of AD animal models, and to promote a consistent cognition improvement in mild-to-moderate AD in humans [59]. Significantly, this probiotic has completed the first Phase III clinical trial in China [189]. In 2020, the U.S. Food and Drug Administration (FDA) gave a formal nod to commence a Phase III clinical trial in the United States to test the drug GV-971 on patients with AD.

## 5. The Endotoxin Hypothesis

*This hypothesis rests on accumulated evidence highlighting the role of LPS in the pathogenesis of neurodegenerative diseases [12]. Known since the end of the XIX century, endotoxins determine inflammation and toxicity [190]. Endotoxins are a common component of the Gram-negative plasma membrane, located in the external layer. Endotoxins can be released following bacterial death or as external membrane vesicles. High levels of Gram-negative bacteria, containing and producing endotoxins, are found in the lower mammalian intestine [191]*.

*The endotoxins, once released, manifest significant differences in their biological activity based on the properties of the lipophilic lipid A portion. In particular, the presence of 6-acyl chains makes the molecule particularly aggressive [192]. Several species of Gram-negative bacteria produce LPS, but the greatest producer of the 6-acyl chain is E. coli, which produces a great amount of Aβ [12]. All the endotoxins bind to the MD2/TLR4 receptor (a complex of myeloid differentiation factor 2 and Toll-like receptor 4); however, while the 6-acyl chains variant strongly activates it, inducing an intense inflammatory response, the 4- or 5-acyl chains act as antagonists on the same receptor. Endotoxins, for their chemical properties, cross the plasma membranes, enter the intestinal cells and, bound to albumin or HDL or chylomicrons, reach the blood stream and the brain. Small amounts of plasmatic endotoxin are detected in all healthy humans; however, higher levels of these molecules have been constantly found in PD, AD, and motor neuron diseases. Indeed, high levels of endotoxin in the gut and brain have been shown to impair the IB and BBB integrity because of local inflammation, and to favor the accumulation of other potential toxic molecules such as Aβ, α-synuclein and some amino acids [59]. Ultimately, high levels of endotoxin also promote the production or aggregation of Aβ, tau protein and α-synuclein in the brain [12].*

*In the brain, endotoxins target microglia selecting the pro-inflammatory M1 phenotype, and astrocytes. M1 and astrocytes produce a high quantity of iNOS and cytokines via the activation of the TLR4 and phagocyte death neurons or even stressed-but-viable neurons through the mechanism of phagoptosis [12,193]. It has not yet been established whether endotoxins prime microglia to neurodegenerative stimuli or vice versa [194]. There is clinical evidence that systemic inflammation accelerates cognitive decline in AD patients. In summary, it is reasonable to assume that any intervention aimed at preventing or treating dysbiosis (reducing the production of toxic molecules) could interrupt or at least slow down the vicious circle endotoxins–neuroinflammation–neurodegeneration [12].*

## Abbreviations

Aβ	amyloid-beta
AHRs	aryl hydrocarbon receptors
AQP4	aquaporin4
BBB	blood–brain barrier
CM	chylomicrons
CNS	central nervous system
CXCR3	CXC chemokine receptors3
EECs	entero-endocrine cells
ENS	enteric nervous system
HDAC1/2	histone deacetylases1/2
IB	intestinal barrier
IL	interleukin
ILC	innate lymphoid cells
iNOS	inducible nitric oxide synthase
iPSC	induced pluripotent stem cell
IS	immune system
LOAD	late onset AD
LPS	lipopolysaccharides
MAMPs	microbe-associated molecular patterns
NO	nitric oxide
NVU	neurovascular unit
PAMPs	pathogen-associated molecular patterns
P2RY12	purinergic 2 receptor Y12
ROS	reactive oxygen species
SCFA	short chain fatty acid
Th1	T helper 1
TLR	Toll-like receptor
TNF	tumor necrosis factor
